# Fecal microbiota transplantation for the treatment of intestinal and extra‐intestinal diseases: Mechanism basis, clinical application, and potential prospect

**DOI:** 10.1002/btm2.10728

**Published:** 2024-11-07

**Authors:** Dongxin Yi, Tao Li, Yuji Xiao, Xue Zhang, Qiangqiang Hao, Feng Zhang, Tianming Qiu, Guang Yang, Xiance Sun, Ying Dong, Ningning Wang

**Affiliations:** ^1^ School of Public Health, Dalian Medical University Dalian China; ^2^ Department of Cardiology The First Affiliated Hospital of Dalian Medical University Dalian China; ^3^ Bishan Hospital of Chongqing Medical University Chongqing China; ^4^ The Second People's Hospital of Dalian Dalian China; ^5^ Occupational and Environmental Health Department School of Public Health, Dalian Medical University Dalian China; ^6^ Global Health Research Center Dalian Medical University Dalian China; ^7^ Department of Nutrition and Food Hygiene School of Public Health, Dalian Medical University Dalian China; ^8^ Department of Gastroenterology The Second Affiliated Hospital of Dalian Medical University Dalian Dalian China

**Keywords:** fecal microbiota transplantation, gut microbiota, intestinal microbiota, intestinal diseases, extra‐intestinal diseases

## Abstract

To review the theoretical basis and therapeutic effects of fecal microbiota transplantation (FMT) in various diseases in animal experiments and clinical studies, as well as the limitations and current standards of FMT application. PubMed and Web of Science databases were searched for articles published only in English between 1975 and 2023 on reliable results of animal experiments and clinical treatment of FMT. The properties of the gut microbiota and its interactions with the host metabolism are critical to human health, and microbiome disturbance is closely associated with human intestinal and extra‐intestinal diseases. Therefore, therapeutic tools targeting on the modulation of gut microbiota have attracted increasing attention, among which FMT represents the most widely studied intervention strategy. This review gathered and summarized application of FMT in intestinal diseases, metabolic diseases, hypertension, cancer, nervous system diseases and arthritis, and elaborated the beneficial effects that can be achieved by altering the microbiota with FMT and the mechanisms of action. In addition, the potential risks and side effects of FMT approach are discussed, as well as current efforts to standardize the development of FMT. Through a systemic review of the outcome and mechanism of FMT in the treatment of intestinal diseases and extra‐intestinal diseases, we aimed to provide a theoretical basis for the construction of an optimized FMT framework, so as to better exert its application prospects.

**Abbreviations**
5‐HT: 5‐hydroxytryptamine
ACPAs: anti‐citrullinated peptide antibodies
ALS: amyotrophic lateral sclerosis
AS: ankylosing spondylitis
ASCVD: atherosclerotic cardiovascular disease
ASD: autism spectrum disorder
BCAA: branched‐chain amino acids
BP: blood pressure
CLDs: chronic liver diseases
FBG: fasting blood glucose
FDA: Food and Drug Administration
FMT: fecal microbiota transplantation
FMT‐AID: FMT and anti‐inflammatory diet
FXR: farnesoid X receptor
GC: glucocorticoid
GI: gastrointestinal
GIQLI: Gastrointestinal Quality of Life Index
GLP‐1: glucagon‐like peptide‐1
GPR: G‐protein coupled receptor
HbA1c: hemoglobin A1c
HDL‐c: high‐density lipoprotein cholesterol
HE: hepatic encephalopathy
ICI: immune checkpoint inhibitors
IL‐6: interleukin‐6
ILG: isoliquiritigenin
IMC: immune‐mediated colitis
LDL‐c: low‐density lipoprotein cholesterol
LPS: lipopolysaccharide
LSI: lifestyle intervention
MS: metabolic syndrome
nCRT: neoadjuvant chemoradiotherapy
NG: nasogastric
NJ: nasojejunal
PAC‐SYM: Patient Assessment of Constipation Symptom Questionnaire
PD: Parkinson's disease
PD‐1: programmed cell death protein 1
Pg: *Porphyromonas gingivalis*
PsA: psoriatic arthritis
PSP: progressive supranuclear palsy
RA: rheumatoid arthritis
SCCAI: Simple Clinical Colitis Activity Index
SCFAs: short‐chain fatty acids
SMT: standard medical therapy
T1DM: type 1 diabetes mellitus
T2DM: type 2 diabetes mellitus
TET: colonic transendoscopic enteral tubing
TMAO: trimethyl‐amine‐N‐oxide
UC: ulcerative colitis
UCEIS: Ulcerative Colitis Endoscopic Index of Severity
WMT: washed microbiota transplantation

## 1 INTRODUCTION

A complex ecosystem in human gut microbiota is formed by 100 trillion microbes, including bacteria, viruses, archaea, fungus, and protists. Their metabolism and interactions are essentially involved in the pathophysiological processes of host organisms and even guide host metabolic and immune pathways. At present, through accurate and perfect high‐throughput sequencing and bioinformatics technology, a variety of intestinal and systemic diseases have been revealed to be caused by the dysbiosis of the human gut microbiota,^1^, ^2^ such as irritable bowel syndrome,^3^ inflammatory bowel disease,^4^ obesity, metabolic syndrome (MS),^5^ neurological and psychiatric disorders^6^ and various cancers, and so forth.^7^ In the 20th century, it was hypothesized that restoration of microbial composition could treat diseases led by marked turbulence of the gut microbiota, and oral administration of probiotics containing desired beneficial microbes is a promising therapeutic approach to re‐establish gut microbiota homeostasis. Probiotics are “active microorganisms capable of conferring health benefits to the host when ingested in enough quantities.” Probiotic products, known as microecological agents, play an immunomodulatory role by affecting Th 1/Th 2 balance, Th 17/Treg balance, B cells, and other adaptive immune cells (including follicular helper T cells and γδT cells) and innate immune cells (including macrophages, neutrophils, and mast cells).^8^ At present, *Lactobacillus*, *Bifidobacterium*, *Escherichia coli*, *Enterococcus* and some yeasts are widely studied and developed.^9^ They are used to remedy intestinal microbiota disorder, improve intestinal barrier function, remove pathogenic microorganisms and potential allergic antigens, prevent secondary bacterial infection, and enhance human immunity. However, the effectiveness of probiotics is influenced by multiple factors, such as the diversity and function of probiotics, the spectrum of their fermentation products, and the environment and adaptability of their survival.^10^

An alternative strategy is FMT, which in fact, is not a new treatment. In the Eastern Jin Dynasty (AD 317–420), Ge Hong recorded in his “Urgent Prescription for Elbow Reserve” a treatment for diarrhea and food poisoning called “Huanglong Soup,” which was similar to FMT.^11^ Currently, FMT has been optimized in the health care setting to be odorless and tastless for the recipient and is delivered by various modalities, including daily oral administration of capsules containing freeze‐dried microorganisms, small bowel infusions through a naso‐duodenal tube, esophago‐gastroduodenoscopy, colonoscopy, or colon retention enema. Similar to probiotics agent, FMT can enrich microbial diversity, rebuild intestinal homeostasis, and reconstruct immunologic function. Gut microbiome provided by healthy donors can (1) compete or antagonize pathogenic bacteria, stimulate the differentiation of intestinal mucosal epithelial cells, promote the repair of intestinal mucosal damage, inhibit apoptosis of intestinal epithelial cell, and thus maintain intestinal epithelial integrity^12^; (2) stimulate the development of mucosal lymphoid tissue and keep the immune system moderately active^13^, ^14^; (3) synthesize anti‐inflammatory factors to reduce inflammatory response^15^, ^16^; (4) decompose fiber and polysaccharide, synthesize protective compounds to participate in host metabolism^17^; (5) restore metabolism of secondary bile acid.^18^

Collectively, the gut microbiota can directly or indirectly interact with the host in a beneficial or harmful manner, or exert specific effects on host health under specific conditions. The balanced microbiome of healthy donors obtained by FMT can regulate the gene expression of intestinal mucosal cells, intestinal mucosal immune function, intestinal ecological environment, and host metabolism, thereby ameliorating the body's regulatory immune response, inflammatory response, and the number and activity of neurotransmitters, and then affect the pathogenesis of intestinal and extra‐intestinal diseases.

For this, Pubmed and Web of Science databases were searched for articles published only in English between 1975 and 2023 on reliable research results of animal experiments and clinical treatment of FMT. The terms we used for retrieving articles were “fecal microbiota transplantation,” “FMT,” “gut microbiota,” “intestinal microbiota,” “intestinal disorders,” “ulcerative colitis,” “immune checkpoint inhibitors (ICI)‐associated colitis,” “constipation,” “metabolic syndrome,” “diabetes,” “hepatic encephalopathy,” “hypertension,” “cancer,” “neurological disorders,” “arthritis,” “adverse effects,” “donor sources,” “delivery methods,” with the strategies as follows: ((fecal microbiota transplantation[Title/Abstract]) OR (FMT[Title/Abstract])) AND (((((((((((((gut microbiota[Title/Abstract]) OR (intestinal microbiota[Title/Abstract])) OR (intestinal disorders[Title/Abstract])) OR (ulcerative colitis[Title/Abstract])) OR (Immune checkpoint inhibitors (ICI)‐associated colitis[Title/Abstract])) OR (constipation[Title/Abstract])) OR (metabolic syndrome[Title/Abstract])) OR (diabetes[Title/Abstract])) OR (hepatic encephalopathy[Title/Abstract])) OR (hypertension[Title/Abstract])) OR (cancer[Title/Abstract])) OR (neurological disorders[Title/Abstract])) OR (arthritis[Title/Abstract])); ((fecal microbiota transplantation[Title/Abstract]) OR (FMT[Title/Abstract]) AND (((adverse effects[MeSH Terms]) OR (donor sources[MeSH Terms]) OR (delivery methods[MeSH Terms]).

## 2 FMT FOR INTESTINAL DISEASES

### 2.1 Ulcerative colitis (UC)

Ulcerative colitis is a piece of illness caused by excessive inflammation resulted from imbalance of intestinal microbiota and mucosal immunity.^19^ The intestinal microbiome of patients with UC is different from that of healthy people, mainly manifested as the reduction of the microbial diversity, such as a decrease in bacteria of *Clostridium* group XI and Va.^20^ The microbiota variation may be an initial pathogeny leading to the occurrence of UC by promoting local and systemic immune response, intestinal mucosal morphological structure damage, and increased permeability.^21^, ^22^, ^23^, ^24^ Therefore, it has been suggested that UC can be alleviated by regulating the composition of microbiota.^25^, ^26^ In a very recent study, fecal bacteria from mice treated with Banxia Xiexin decoction, a classical Chinese medicine formula, was found to improve the balance and diversity of intestinal microbiota in UC mice, and to regulate amino acid, purine and lipid metabolism, showing a positive therapeutic effect on UC mice.^27^

Tkach et al. evaluated the efficacy, safety, and tolerability of FMT in patients with mild and moderate UC in a randomized clinical trial. UC patients were randomly assigned to two groups: one group (*n* = 27) was given mesalazine as the standard treatment group; subjects in the FMT group (*n* = 26) received fresh material from healthy donors along with the indicated dose of mesalazine as adjuvant therapy. After 4 and 8 weeks of treatment, the clinical efficacy of the two approaches was assessed. A clinical response was detectable in both groups, as evidenced by a remarkable decrease in the frequency of bowel movements and a gradual normalization of stool consistency after 4 weeks (*p* = 0.583), with clinical response rate 16/26 (61.5%) in the FMT group and 14/27 (51.9%) in the standard treatment group. After 8 weeks, 84.6% of the sicks in the FMT group achieved remission of UC, mainly characterized by partial Mayo score ≤2 and a decrease in fecal calprotectin level. Although there was no statistical difference compared with remission rate of 70.4% in the standard‐therapy group (*p* = 0.215), the Mayo score of the FMT group decreased more significantly (1.34 ± 1.44 vs. 2.14 ± 1.4; *p* = 0.045). In addition, favorable changes in microbiome composition in FMT group was more pronounced as the levels of Bacteroidota (Bacteroidetes) and Bacillota (Firmicutes) almost returned to normal and the significant increase in microbial abundance compared with baseline was occurred only in the FMT group (from 23.0% to 31.5%, *p* < 0.05). Moreover, butyrate‐producing *Faecalibacterium prausnitzii* showed a significant increase in abundance after FMT, which may also indicate an improvement of the gut microbiota. In conclusion, the findings show that FTM is a well‐tolerated, effective, and safe treatment for patients with mild and moderate UC compared with mesalazine therapy.^28^

Similarly, Huang et al. compared the safety and curative effect of FMT with glucocorticoid (GC) induction in UC in a prospective cohort study. Twenty‐two patients were divided into two groups, with 62 patients receiving FMT and 60 receiving GCs. At week 12, 54.8% of the FMT group (*n* = 34) and 48.3% of the GC group (*n* = 29) reached the primary outcome of clinical and endoscopic relief (*p* = 0.30). There were 35 cases of adverse events in GC group and 14 in FMT group, the former including 2 serious adverse events, indicating an obviously higher incidence of adverse events in GC group than in FMT group.^29^

Kedia et al. randomly assigned the enrolled 66 UC patients to the FMT, anti‐inflammatory diet (FMT‐AID) group and the optimized standard medical therapy (SMT) group in equal proportions over 1 year. The FMT‐AID group received fecal suspension from multiple healthy donors that were infused 7 times per week via colonoscopy infusions, accompanied by an anti‐inflammatory diet, whereas patients in the SMT group received baseline medication alone. Patients who had a clinical response [Simple Clinical Colitis Activity Index (SCCAI) <2, and Ulcerative Colitis Endoscopic Index of Severity (UCEIS) <1] or remission at week 8 were observed for another 40 weeks until week 48. At week 8, the clinical response rate (23/35, 65.7%), remission rate (21/35, 60%) and deep remission rate (12/33, 36.4%) in FMT‐AID group were all significantly higher than those in SMT group [(11/31, 35.5%), 10/31 (32.3%) and 2/23 (8.7%), respectively]. At week 48, the overall clinical remission/response rate of FMT‐AID group was higher than that of SMT group [50% (15/30) vs. 32.3% (10/31), *p* = 0.16]. By week 48, FMT‐AID was superior to SMT in keeping deep remission (6/24 (25%) vs. 0/27, *p* = 0.007). The results proved that multi‐donor FMT was valid in contributing to deep relief of mild and moderate UC, which was sustained for over 1 year on an anti‐inflammatory diet.^30^

Overall, FMT is tolerable and effective in the clinical remission of mild to moderate UC, with less side effects than GC induction and more benefits than aminosalicylic acid drugs alone, and long‐term effects are expected when combined with an anti‐inflammatory diet.

### 2.2 Immune checkpoint inhibitors (ICIs)‐associated colitis

Immune checkpoint inhibitors benefit many patients with melanoma and other cancers but it can cause adverse events, such as ICI‐colitis, and hitherto, the pathogenesis of ICI‐colitis remains unclear. Gut microbiota is considered to be an important mediator of ICI toxicity and efficacy by affecting host immune system and anti‐tumor immunity.^31^

The typical treatment for ICI‐associated colitis is immunosuppressive therapy with medications. A case of successful treatment of anti‐programmed cell death protein 1 (PD‐1) inhibitor‐associated colitis by FMT have been reported. In a patient with malignant melanoma of the palate who developed diarrhea, GI bleeding, and other symptoms after the third PD‐1 treatment, which persisted after treatment with glucocorticoids and two doses of anti‐integrin, execution of three consecutive FMT sessions contributed to rapid remission with complete resolution of the above symptoms.^32^

Wang et al. performed FMT in two cancer patients hospitalized for diarrhea/colitis after CTLA‐4 and PD‐1 combined blockade treatment. The abundance and composition of bacteria in stool samples before and after the intervention were assessed by 16S rRNA sequencing, and in both patients, the number of observed OTUs increased after each FMT, although in both cases they were later destabilized. In particular, *Bacteroidia* and *Verrucomicrobiae* were significantly deficient, and *Akkermansia* was significantly more abundant after FMT in the first patient. The abundance of *Blautia* and *Bifidobacterium* increased obviously in the second patient after FMT, while the abundance of *Escherichia* increased and *Bacteroides* decreased after the second FMT. This small cohort study was supported by data from another large case series of 12 patients with refractory immune‐mediated colitis (IMC), in which FMT was effective in IMC patients with a significant increase in alpha diversity and the abundance of *Collinsella* and *Bifidobacterium*.^33^ These cases provide new evidence that FMT can effectively and rapidly improve refractory ICI‐associated colitis by modifying the gut microbiome.^34^

### 2.3 Constipation

Chronic constipation, characterized by low‐grade mucosal inflammation, is a common multifactorial GI disorder whose etiology and pathophysiology remain unclear. Nevertheless, dysregulation of the gut microbiota has been documented in chronic constipation. Khalif et al. performed fecal culture analysis on constipated patients, which showed high levels of *Enterobacteriaceae* (e.g., *E. coli*), *Staphylococcus aureus*, and fungi and low levels of *Bifidobacterium* and *Clostridium* species.^35^ Moreover, Zhu et al. suggested a significant reduction in the level of Bacteroidota (specifically *Prevotella*) in patients with constipation.^36^ In addition to a healthy gut microecology, bacterial metabolites are also important factors for promoting intestinal peristalsis. For example, dietary fiber is broken down by intestinal bacteria to short‐chain fatty acids (SCFAs), which release 5‐hydroxytryptamine (5‐HT), an important metabolite to maintain intestinal function.^37^

FMT is a relatively new tool for clinical treatment of constipation. Ge et al. applied FMT therapy in 6 patients with slow transit constipation for 12 weeks, the frequency of defecation increased significantly from 1.6 ± 0.2 times per week before treatment to 5.0 ± 0.4 times, and fecal viscosity improved from 2.0 ± 0.3 to 3.3 ± 0.2 (*p* = 0.0025), as well as colonic transit time, Patient Assessment of Constipation Symptom Questionnaire (PAC‐SYM) and Gastrointestinal Quality of Life Index (GIQLI) scores.^38^ In addition, FMT could enhance the efficacy of retrograde colonic enema, thereby improving the safety and effectiveness of the treatment of intractable childhood constipation.^39^

## 3 FMT FOR EXTRA‐INTESTINAL DISEASES

### 3.1 FMT for MS

MS is a clinical syndrome that multiple cardiovascular risk factors such as obesity, hyperglycemia, hypertension, and dyslipidemia coexist in the same individual, which will significantly increase the risk of diabetes and cardiovascular disease. Nowadays, lifestyle intervention and medication are recommended strategies for MS. To avoid significant side effects brought by long‐term drug therapy, it is of great significance to find treatments with sufficient safety and reliability. Microbe‐derived metabolites, such as SCFAs, bile acids, and trimethyl‐amine‐N‐oxide (TMAO), are known to exert beneficial effects on the host through anti‐inflammatory and antioxidant activities. However, some microbe‐derived metabolites, such as cytotoxins, genotoxins, and immunotoxins are involved in a wide range of pathophysiological processes that are associated with the risk of MS.^40^, ^41^

It has been shown that FMT can restore the deficits observed in animal models of fructose‐induced MS, and reduced markers of oxidative stress and inflammation. Furthermore, the abundance of *Coprococcus* and *Ruminococcus*, which are associated with proinflammatory markers,^42^ were also cut down after FMT treatment.^43^ Isoliquiritigenin (ILG) has been reported as a prebiotic to prevent and treat metabolic disorders. The beneficial action of ILG on metabolic disorders was achieved by augmenting the abundance of bacteria species resistant to metabolic diseases and up‐regulating genes that can improve intestinal barrier function. In an animal study, FMT from ILG‐fed donors counteracts high fat diet‐induced weight gain and visceral adipose tissue mass, expression of genes associated with inflammation, insulin resistance, and glucose intolerance. Therefore, gut microbiota remodeled by prebiotics as a donor of FMT can improve MS.^44^

Mocanu et al. provided evidence that single‐dose oral FMT in patients with MS is safe and well tolerated, and can improve insulin sensitivity.^5^ In a clinical trial evaluating the efficacy of FMT in adolescents with MS, FMT improved insulin sensitivity and glucose metabolism, with a 34% improvement in HOMA‐IR, a 29% reduction in fasting insulin levels, and a 7% decrease in fasting blood glucose (FBG) after 6‐week intervention. Even more gratifying, the resolution rate of metabolic abnormalities at 26 weeks after the intervention was 78%, demonstrating a significant benefit.^45^

Washed microbiota transplantation (WMT), a novel method of FMT, that appends safety measures such as bacteria isolation, rinsing, and quantification of healthy donor feces. Wu et al. included a total of 237 patients with functional bowel diseases receiving WMT treatment, who were allocated into MS group (*n* = 42) and non‐MS group (*n* = 195). Baseline and short‐, medium‐, and long‐term outcomes were collected before WMT, approximately 1, 2, and 6 months after the first WMT cycle. Specifically, WMT markedly improved the overall outcomes of MS patients, with 40.48% of MS patients achieved short‐term recovery (*p* < 0.001), 36.00% achieved medium‐term recovery (*p* = 0.003), and 46.15% achieved long‐term recovery (*p* = 0.020). In short term outcomes, a significant reduction in FBG, triglyceride, systolic blood pressure (BP) and BMI, and increased high‐density lipoprotein cholesterol (HDL‐c) were observed. In the middle stage, FBG, total cholesterol, low‐density lipoprotein cholesterol (LDL‐c), non‐HDL and BMI were notably reduced. According to the risk stratification of atherosclerotic cardiovascular disease (ASCVD), the patients were further divided into very high‐risk group, high risk group, moderate risk group and low risk group. In the MS high‐risk group, WMT showed an obvious short‐term (*p* = 0.029) and medium‐term (*p* = 0.011) ASCVD de‐escalation effect on MS patients. Furthermore, gut microbiota composition from the phylum to genus level showed improved gut microbiota in patients with MS after WMT.^46^

Together, these studies evidence provide an optimistic effect of FMT in improving metabolic characteristics of MS, and in particular, as the modified means of FMT, WMT shows an outstanding efficacy.

### 3.2 FMT for diabetes

Gut microbiota plays a significant role in the occurrence and development of type 2 diabetes mellitus (T2DM), potentially through molecules such as SCFAs, lipopolysaccharide (LPS) and branched‐chain amino acids (BCAA), which are affected by intestinal mucosal barrier feature and intestinal inflammation levels.^47^ SCFAs can stimulate enterocytes to secrete glucagon‐like peptide (GLP)‐1 through receptors G‐protein coupled receptor (GPR) 41 and GPR43, thereby modifying pancreatic function and insulin release.^48^ According to previous studies, reduced relative abundance of gut butyricogenic bacteria and impaired butyrate production was exhibited in T2DM patients, as well as increased levels of LPS; further, the overgrowth of conditionally pathogenic *Enterobacteriaceae* can be attributed to the proinflammatory function of hydrogen sulfide and transport function of BCAA.^49^ In addition, the gut microbiome has been found to play a role in the pathogenesis of inflammation‐induced gestational diabetes mellitus.^50^

Previous study with a mouse model of T2DM induced by high‐fat diet combined with streptozotocin showed that FMT could improve insulin resistance, repair damaged islets, and inhibit apoptosis of islet β cells.^51^ In a 12‐week placebo‐controlled trial, Ng et al. randomized 61 obese subjects with T2DM to one of three parallel groups: FMT plus lifestyle intervention (LSI), FMT alone, or sham transplantation plus LSI every 4 weeks. The proportion of microbiota obtained from lean donors ≥20% detected in subjects by fecal metagenomic sequencing at week 24 was the primary outcome. The percentage of subjects achieving the primary outcome at week 24 was 100% in the FMT + LSI group, 88.2% in the FMT alone group, and 22% in the sham‐LSI group (*p* < 0.0001). Besides, FMT with or without LSI increased butyrate‐producing bacteria, whereas, FMT + LSI increased the number of *Bifidobacterium* and *Lactobacillus* (both *p* < 0.05) compared with FMT alone. Beneficial changes were also emerged in lipid profile and stiffness of the liver (both *p* < 0.05). In conclusion, repeated FMT enhanced the efficacy and duration of efficacy in T2DM patients.^52^ A recent study evaluating the clinical reaction of 17 T2DM patients to FMT showed significant improvement in T2DM symptoms in 11 patients, mainly manifested by statistically conspicuous diminish in hemoglobin A1c (HbA1c), fasting and postprandial glucose, uric acid, while postprandial C‐peptide (an indicator related to serum insulin) increased. Moreover, individuals with a high abundance of anaerobes, which is associated with increased insulin resistance, showed a preferable clinical response to FMT intervention.^53^

Type 1 diabetes mellitus (T1DM) is an autoimmune disease characterized by progressive β‐cell destruction. The interaction between gut microbes and the innate immune system is a key factor in the origin of T1DM,^54^ and alterations in intestinal epithelial barrier function can also contribute to the development of T1DM.^55^ Likewise, some studies have revealed beneficial effects of FMT in T1DM patients. FMT stabilized residual β‐cell function in subjects with newly diagnosed T1DM and prevented the decline in endogenous insulin production for up to 12 months after onset.^56^ Xie et al. reported a case of T1DM patient who suffered from poor glycemic control after insulin therapy, and developed GI symptoms of recurrent nausea and vomiting, and intractable constipation; while the patient had a good clinical response to FMT, especially the nausea and vomiting symptoms were obviously relieved. There were also favorable shifts in nutritional status, constipation, and glycemic control (FBG, HbA1c). Meanwhile, the microbial community structure and composition of patients after FMT was similar to that of healthy donors, but not at all before treatment.^57^ In a T1DM mouse model, FMT was found to markedly reduce blood glucose and augment *Lactobacillus* species in the small intestine.^58^

In addition, transplantation of grafts made with fecal microbiota from healthy donors has been shown to significantly ameliorate distal symmetric polyneuropathy and glycemic variability in patients with diabetes by modulating the composition and function of gut microbiota.^59^, ^60^

On the whole, FMT improves T2DM phenotypes by modulating inflammatory metabolites of the gut microbiota, but evidence for disease progression and risk in T1DM is limited.

### 3.3 FMT for hepatic encephalopathy (HE)

The crosstalk between the gut and liver is carried out through intestinal barrier, intestinal microbiota, intestinal immune system, portal vein and other elements, namely the gut‐liver axis. Therefore, the disturbance of gut‐liver axis is the common pathophysiological basis of various liver diseases.^61^ In chronic liver diseases (CLDs), reduced bile flux causes cholestasis, which impairs liver‐intestinal circulation and primarily affects the microbiota, so a diminish in microbial richness and diversity has been observed in different types of CLDs.^62^ Liver cirrhosis is the end stage of CLD with different etiologies and is also closely related to the reduction of intestinal bile acid secretion. Bile acid is a strong regulator of Farnesoid X receptor (FXR)‐axis, which is essential for maintaining the homeostasis of intestinal epithelial barrier and gut‐vascular barrier, thus its decreased secretion promotes bacterial translocation and then leads to decompensation of cirrhosis.^63^, ^64^ What's more, the microbiome of patients with cirrhosis varied with the severity of the condition. Specifically, in cirrhosis, a significant decrease in helpful commensal bacteria such as *Ruminococcaceae* and *Trichomonadidae*, *Clostridium XIV*, and *Lachrillaceae*, while an increase in pathogenic taxa such as *Streptococcaceae* and *Enterobacteriaceae*, *Enterococcaceae*, S*taphylococcaceae* was observed.^65^, ^66^ HE is a serious complication of liver cirrhosis and is mainly induced by factors related to disordered intestinal environment, such as infection, GI bleeding, endotoxemia, and harmful changes in gut microbiota, and so forth.^67^ For instance, an augment in the relative abundance of ammonia‐producing bacterial species will trigger the onset of HE.^68^

FMT has a confirmed effect in reducing serum ammonia generation and reducing the occurrence of HE in several animal models.^69^ With applying the inhibitory control test and the Stroop test, which weekly assesses subjective and objective changes in mental status, Kao et al. have proved for the first time that persistent FMT contributes to cognitive improvement in mild HE.^70^ Bloom et al. evaluated the safety and therapeutic effect of FMT in patients with HE and, for the first time, investigated the donor and recipient factors that influence the efficacy of FMT.^71^ In a phase I, placebo‐controlled trial, Bajaj et al. randomized 20 enrolled patients with recurrent HE in a 1:1 ratio to receive either 15 FMT capsules from a single donor enriched in the families *Tricspira* and *Ruminococcaceae*, or placebo. Clinical follow‐up was performed and the standard care was maintained up to 5 months. FMT was associated with improved alpha diversity of duodenal bacteria, dysbiosis, EncephalApp (A smartphone app detects occult hepatic encephalopathy in patients with cirrhosis) performance. After FMT, the expression of anti‐microbial peptide, E‐cadherin and defensin A5 improved whereas the expression of interleukin (IL)‐6 and LPS binding protein decreased, accompanied by increased abundance of *Ruminococcaceae* and *Bifidobacteriaceae* in the duodenum, while a decrease in *Streptococcaceae* and *Veillonellaceae* taxa, as well as *Veillonellaceae* in the sigmoid colon and stool. Even more encouraging, there was a trend toward lower hospitalization rates among patients randomized to FMT.^72^ Overall, these findings suggest that FMT is safe and tolerated for improving symptoms in patients with cirrhosis and recurrent HE, thereby reducing the tendency to be hospitalized.

### 3.4 FMT for hypertension

Hypertension is the most common cardiovascular disease, affecting about 30% of the global adult population.^73^ Earlier studies showed elevated BP in germ‐free rats, suggesting a subtle role for the gut microbiota in BP adjustment.^74^ Yang et al. compared changes in the gut microbiota in spontaneously hypertensive rats and chronic angiotensin II infusion rat models. It was found that spontaneously hypertensive rats had a significant decrease in microbial richness, diversity and evenness, an increase in Bacillota/Bacteroidota ratio, and a sharp reduction in acetic, butyric and lactic acid‐producing bacteria.^75^ It has been shown that the feces of hypertensive mice exhibit gut dysbiosis featured by a higher Bacillota/Bacteroidota ratio, lower levels of acetogenic and lactogenic bacteria, as well as lower strict anaerobes. In clinical studies, the microbial composition of individuals with prehypertensive, hypertensive was significantly different from that of their counterparts, with respect to several bacterial taxa.^76^ From a mechanistic perspective, SCFAs produced by gut microbiota have been shown to regulate BP by stimulating the host GPR pathway, which affects renin secretion and BP regulation.^77^ In addition, gut microbiota can participate in the control of BP by affecting the activation of T cells and the accumulation of T cells in blood vessels.^78^ Gut microbiota is likely to affect the pharmacokinetics of antihypertensive drugs through a variety of pathways, including the regulation of bacterial metabolism, bacterial transport, and intestinal transport, suggesting that the therapeutic efficacy of antihypertensive drug can be advanced by using substances that alter the microbiota composition.^79^ Losartan, an antihypertensive drug, can improve intestinal integrity in hypertensive rats by alleviating intestinal dysbiosis and sympathetic nerve drive.^80^ Receiving FMT treatment from donors taking angiotensin receptor blocker could protect against collagen and reactive oxygen species production in blood vessels and attenuate intestinal dysfunction.^81^ A separate meta‐analysis showed significant reductions in both systolic and diastolic BP in patients who daily ingested probiotics of 10^9^ colony‐forming units, providing a foundation for FMT application in hypertension.^82^

Toral et al. have found in an animal experiment that BP responds to the alteration in the gut microbiota through neuroinflammation and sympathetic nervous system activity.^83^ Specifically, FMT reduces BP by enhancing the relaxation effect of acetyl choline on aortic endothelium and decreasing NADPH oxidase activity, accompanied by an augment in the Tregs infiltration and a reduction in the Th 17 cell population in the vessel wall.^79^

Zhong et al. have displayed that rinsed microbiota transplantation (the same as WMT) can significantly lower BP values at discharge in hypertensive patients compared to that at admission (systolic BP change: −5.09 ± 15.51, *p* = 0.009; diastolic BP change: −7.74 ± 10.42, *p* < 0.001). Moreover, the decrease in BP was greater in patients receiving WMT through the lower GI tract and those who had never received antihypertensive drugs. WMT showed a short‐term (3–7 days) rather than medium‐term (1 month) hypotensive function, but its duration of action was longer than that of conventional antihypertensive drugs.^84^ Therefore, manipulation of gut microbiota may provide a new therapeutic strategy for hypertension, possibly by modulating the renin system, sympathetic nervous system and vascular immune system, and FMT may serve as an auxiliary approach to antihypertensive drugs, which should be further affirmed by more clinical trials.

### 3.5 FMT for cancer

Gut microbiota is closely related to the occurrence and development of cancer, and can further interact with cancer therapeutics through pharmacokinetics and pharmacodynamics. Intestinal microecological disorders promote tumorigenesis by altering the host genome, destroying DNA stability, and inducing metabolic disorders, immune dysfunction and barrier damage.^85^, ^86^ Wynder et al. used germ‐free and conventional rats to study the effect of the gut microbiota on the sensitivity of the colon to the carcinogenic effects of 1,2‐dimethylhydrazine, showing that only 20% of germ‐free rats developed colonic tumors, whereas 93% of conventional rats developed multiple colonic tumors.^87^ In smoke expose‐induced tumorigenesis, gut dysbiosis was observed in mice, with enrichment of *Eggerthella lenta* and depletion of *Parabacteroides distasonis* and *Lactobacillus*.^88^ In addition, growing evidence has shown the critical role of gut microbiota in modulating the outcome of cancer treatment, especially in chemotherapy and immunotherapy. Shi et al. investigated the correlation between the gut microbiome and treatment response to neoadjuvant chemoradiotherapy (nCRT) and found that the relative abundance of several bacterial taxa differed in rectal cancer patients before and after nCRT. There were also differences in microbiota composition between responders and non‐responders, with responders enriched in *Shuttleworthia* and non‐responders enriched in *Clostridium*.^89^ Anti‐CTLA‐4 antibody has been successfully applied in cancer immunotherapy, and it has been suggested that the anti‐tumor role of CTLA‐4 is dependent on different *Bacteroides* species, which play a vital function in CTLA‐4‐blocked immune stimulation.^90^ Patients with advanced melanoma can obtain long‐term clinical benefit from PD‐1 therapy.^91^ Studies have indicated that the tumor response to anti‐PD‐1 immunotherapy is also influenced by the gut microbiome.^92^, ^93^, ^94^ However, the modulatory role of microbiota in the patients with cancer has not made prominent progress in clinical trials. In 10 patients with anti‐PD‐1 refractory metastatic melanoma, a phase I clinical trial was conducted to evaluate the efficacy of FMT and re‐induction with anti‐PD‐1 immunotherapy. The results showed significant differences in gut microbiota composition from baseline in all subjects after treatment, with higher abundance of immune‐potent *Veillonellaceae* family and *Trichosporonaceae*, and a smaller relative number of *Bifidobacterium*. Besides, FMT treatment was shown to be associated with profitable mutations in immune cell infiltration and gene expression profiles in the gut lamina propria and tumor microenvironment, such as upregulation of multiple immune‐related genes, such as interferon‐γ‐mediated signaling, T cell activation, MHC class II protein complex, dendritic cell differentiation, and T helper cell type 1 immune responses. All but one unresponsive patient had increased post‐treatment CD68+ infiltration. However, no clear correlation between gut bacterial changes and clinical response to treatment was observed.^95^

Davar et al. assessed the safety and efficacy of response‐derived FMT (from seven donors responsive to pembrolizumab) plus anti‐PD‐1 in patients with PD‐1‐refractory melanoma. This combination proved to be tolerable, with clinical benefit in 6 out of 15 patients, in which responders exhibited increased taxonomy abundance, activation and differentiation of CD8+ T cell, and reduced amount of circulating IL‐8 and IL‐8‐producing myeloid cells, which were associated with improved anti‐PD‐1 response and immune resistance. In conclusion, FMT and anti‐PD‐1 together successfully colonized the gut of responders and altered the microbiota composition, reprogramming the tumor microenvironment to counter anti‐PD‐1 resistance in the PD‐1 advanced melanoma subgroup.^96^ Recently, FMT combined with tislelizumab and fruquintinib was revealed to exert favorable anti‐tumor efficacy and acceptable safety in refractory metastatic colorectal cancer.^97^ Taken together, these early findings imply the great promise of microbiota modulation in melanoma therapy.

### 3.6 FMT for neurological disorders

The gut‐brain‐microbiota axis refers to a two‐way information communication system between the central nervous system and the GI tract through the functional integration of metabolites, hormones and immunomodulators. Bidirectional signaling between the gut microbiota, gut, and brain is realized through neuronal pathways involving the circulatory system, central nervous system, and enteric nervous system.^98^, ^99^ FMT alleviated “plaque disruption,” behavioral changes, cognitive deficits, and brain amyloid‐β (Aβ) deposition in mouse model of Alzheimer's disease.^100^, ^101^ Sun et al. have indicated that FMT protect Parkinson's disease (PD) mice by inhibiting intestinal and neural inflammation and suppressing Toll‐like receptors (TLR)‐4/TNF‐α signaling. Specifically, fecal samples of PD mice showed decreased Bacillota and *Clostridia*, and increased Desulfobacterota (Proteobacteria), *Uricales* and *Enterobacterales*, a disturbed gut microbiota leading to motor deficits and lessened striatal neurotransmitters. FMT intervention not only alleviated intestinal microbial dysbiosis, restored fecal SCFAs, but also improved motor function, aggrandized the amount of dopamine and 5‐HT and their metabolites in the striatum, and recovered the levels of dopaminergic neurons and tyrosine hydroxylase, thereby providing potential neuroprotection in PD mice. In addition, FMT significantly reduced the number of activated substantia nigra microglia and astrocytes, and inhibited the expression of TLR‐4/TNF‐α signaling pathway in the intestine and brain.^102^ DuPont et al. have demonstrated the tolerance to multiple doses of FMT in human PD patients with increased abundance of gut microbes associated with intestinal transport and intestinal motility, and FMT ultimately improves subjective motor and non‐motor symptoms.^103^

Patients with autism spectrum disorder (ASD) are often accompanied by GI problems, including constipation, diarrhea, food allergy and dyspepsia, and the severity of these symptoms is related to behavioral symptoms.^104^ Therefore, gut dysbiosis is suggested to be involved in GI symptoms and neurodevelopmental disorders in ASD, and indeed, the gut microbiome was altered in children with ASD compared with those without ASD.^105^ In a nonrandomized, open‐label clinical trial, 18 children with ASD were treated with 10 weeks of FMT and followed up for 8 weeks. FMT treatment resulted in significant relief of GI symptoms and improvement in ASD‐related behaviors in 80% subjects.^106^ Recently, Li et al. conducted a 12‐week study involving 40 ASD children and 16 counterparts without GI symptoms with significant distinctions in baseline characteristics of behavior, GI symptoms, and gut microbiota. After 8 weeks of FMT treatment, improvements in mood, behavior, language, and affective parameters without any serious complications were observed, as well as GI symptoms, and oral and rectal FMT have similar efficacy. Further, serum 5‐HT and aminobutyric acid concentrations decreased, whereas dopamine levels increased after 4‐week of FMT. Donor microbial colonization was detected after FMT, and decline in *Eubacterium coprostanoligenes* abundance was positively correlated with the alleviation of GI symptoms, suggesting that FMT may improve GI and behavioral symptoms in patients with ASD by adjusting the gut microbiota, especially by targeting *Eubacterium coprostanoligenes*.^107^

By conducting FMT on mice, Liu et al. found that gut microbes from patients with inflammatory depression could cause the mice to exhibit depression and anxiety‐like behaviors, along with increased levels of inflammatory factors in serum and brain and disruption of the gut barrier, suggesting that the gut microbiome may be involved in the neuroinflammation of inflammatory depression, and the gut microbiota may be a therapeutic target for inflammatory depression.^108^ In a recent report, FMT was regarded to be effective in the treatment of depression. Doll et al. described two patients with major depressive disorder who primarily received oral frozen FMT capsules as add‐on therapy, with both patients experiencing improvement in depressive symptoms and GI symptoms 4 weeks after transplantation, and the advantage persisted for 8 weeks in one patient. However, the effect of FMT intervention on taxon abundance varied between patients.^109^

Amyotrophic lateral sclerosis (ALS) is an advanced neurodegenerative disease associated with age, genetic and environmental factors, as well as the gut microbiota and its metabolites. In a mutant superoxide dismutase 1 (SOD1^G93A^) familial ALS mouse model, turbulence in the gut microbiota were observed, including diminished abundance of *Akkermansia*, *Coriobacteriaceae*, and *Adlercreutzia*, which may affect mouse survival.^110^ Modulating gut microbiota by FMT is expected to be a feasible strategy for the treatment of ALS, however, experimental data on patients are limited. Lu et al. released the first case report on the use of WMT for ALS, stating that WMT can prevent amyotrophic lateral sclerosis from worsening.^111^ Similarly, another case report has revealed that FMT may ameliorate respiratory failure in amyotrophic lateral sclerosis by adjusting the gut microbiota, including increased levels of the beneficial bacteria *Bacteroides* and *Faecalibacterium prausnitzii*, as well as improved metabolism, for example by participating in arginine biosynthetic metabolites.^112^ In a phase 2, single center, randomized clinical trial, FMT treatment was reported to reduce intestinal inflammation and improve intestinal barrier damage in progressive supranuclear palsy (PSP)‐Richardson's syndrome (PSP‐RS) by correcting gut dysbiosis. Notably, it significantly improved motor and non‐motor symptoms in PSP‐RS patients.^113^

Further exploration of the relationship between intestinal microbiota and nervous system diseases may advance the diagnosis and treatment of diseases. Based on the therapeutic mechanism of restoring gut microbiome homeostasis, FMT as a treatment strategy for nervous system diseases is likely to bring new hope to patients.

### 3.7 FMT for arthritis

Rheumatoid arthritis (RA) is an illness caused by immune dysfunction, and its pathogenesis involves both genetic and environmental elements. In an animal trial, oral infection with *Porphyromonas gingivalis* (Pg) was reported to induce gut microbiota dysregulation and joint destruction.^114^ It has been further demonstrated that Pg affected the disease activity of RA patients by inducing the production of anti‐citrullinated peptide antibodies (ACPAs) and the inflammatory cascade. Colonization of Pg in the gut and its toxin Pg‐LPS may alter the gut microbial composition, resulting in gut dysbiosis, intestinal barrier disruption, and subsequent transfer of live bacteria or their metabolites into the bloodstream.^115^ Further, both diversity and species richness of the microbiome were much less in RA patients than in healthy individuals.^116^ The study by Chiang and colleagues documented lower abundance and evenness of gut microbial taxa in RA patients contrasted to healthy subjects, with higher abundance of *Verrucomicrobiae* and *Akkermansia*. In particular, microbiota alpha diversity was also significantly different between autoantibody‐positive and autoantibody‐negative patients.^117^ It has been reported that antibacterial drugs such as minocycline and sulfasalazine are effective in some patients with RA, and it can be deduced that the gut microbiota is highly involved in the development of the disease, and FMT may be a valuable tool.

Zeng et al. presented a case of RA treated with FMT. Fecal microbiota suspension prepared from 8‐year‐old healthy children's feces was injected into the colon of RA patients through colonoscopy. On day 7 after FMT, Health Assessment Questionnaire Disability Index reduced sharply from 1.4 to 0.05. On days 42 and 78 after FMT, Disease Activity Score 28 was decreased from 1.9 to 1.4, and rheumatoid factor titers were dropped from 158 to 135.9 IU/mL. What's more encouraging was the reduction in the dose of etanercept, dropping from 50 mg/week to 25 mg/10 days after FMT.^118^ It is suggested that FMT may be an optional treatment strategy for RA, while evidence from intervention trials is urgently needed.

A patient with refractory ankylosing spondylitis (AS) improved significantly after one FMT and further recovered after two additional FMT,^119^ expanding the therapeutical prospect of FMT in arthritis. The effects of gut microbiota on AS are mediated through intestinal inflammation, mucosal Th17 expansion, and lymphocyte migration. In patients with psoriatic arthritis (PsA), Kragsnaes et al. investigated the effect of FMT on inflammation‐associated plasma proteins, and found that three proteins (TNF, IFN‐γ, and SLAMF1) were significantly different between patients who received FMT and sham transplants, implying that FMT may induce a systemic immune response to PsA.^16^

A summary of the mechanisms by which FMT restores changes in intestinal diseases, metabolic diseases, hypertension, cancer, nervous system diseases, and arthritis is presented in Graphical Abstract.

## 4 MANAGEMENT OF FMT

Conventional FMT consists of three major steps: identification of the donor, preparation of fecal filtrate and transplantation to the patient. The selection of appropriate donor is the key to the success of FMT, and there are two main sources of donor feces: patient‐directed donation and stool bank.^120^ At present, the use of universal donors as FMT donors has become a consensus, that is, young donors with normal BMI need to be selected and subjected to rigorous screening, including a medical history collection (chronic disease, neurologic disease, HIV, blastocyst, and poliovirus, etc.), serologic testing, and screening for fecal parasites, virologic, and bacterial pathogens, to minimize the potential risk of transmission of infection or microbiome‐related disease.^121^ It has been suggested that the use of feces from multiple healthy donors to enrich the microbiota composition may enhance the efficacy of FMT, whether by infusion or ingestion.^122^ Currently, stool banks to provide fecal materials for FMT have been established in many countries, greatly reducing the cost of FMT application and ensuring the efficacy and safety of FMT, such as OpenBiome approved by United States Food and Drug Administration (FDA).^123^

**FIGURE 1:**
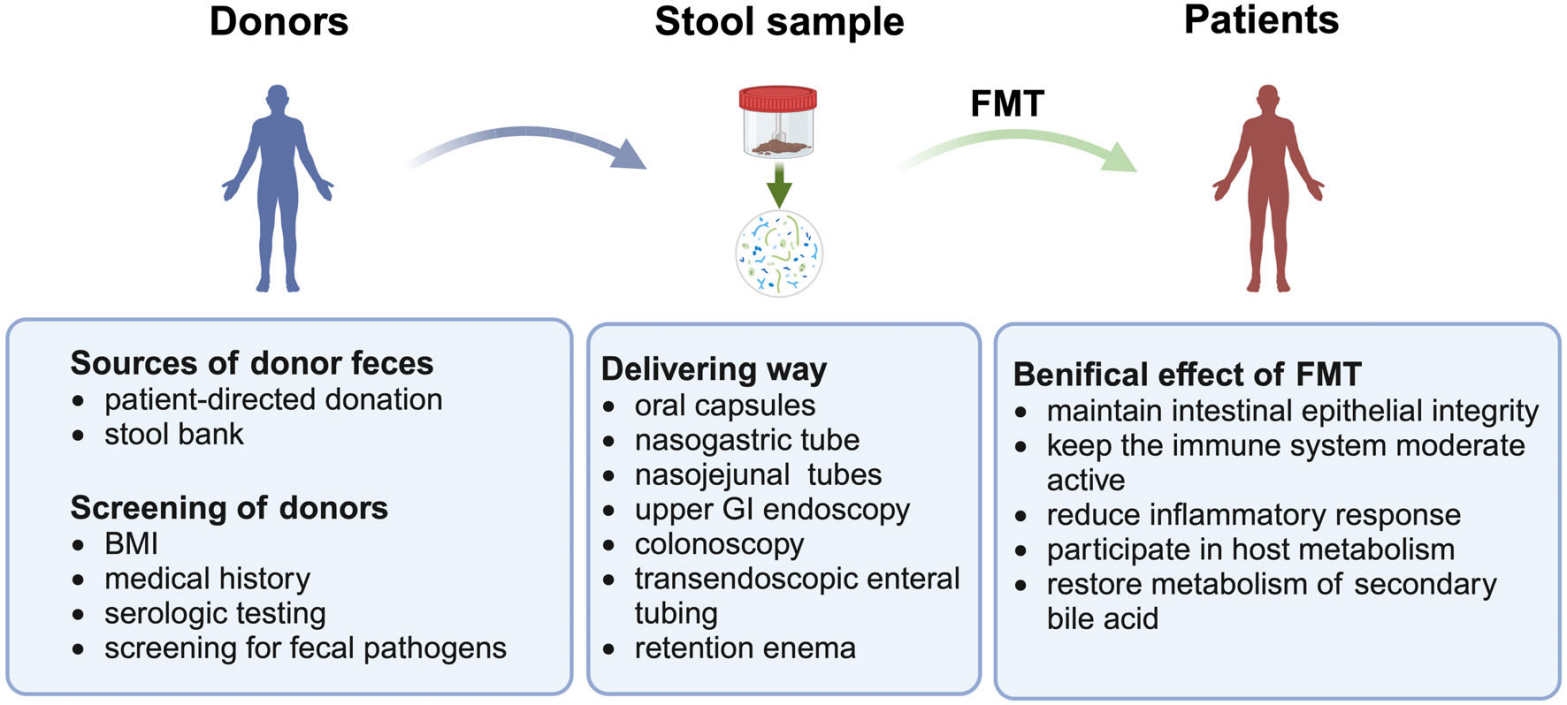
General procedures for fecal microbiota transplantation (FMT).

With regard to the clinical application of FMT, patients can now be managed with a combination of the upper and lower GI approach, that is, oral capsules, nasogastric (NG) tubes and nasojejunal (NJ) tubes, or upper GI endoscopy, colonoscopy, retention enema and colonic transendoscopic enteral tubing (TET), each of which has advantages and disadvantages. NG tubes, for example, require no sedation, cost less, and cause minimal trauma to the patient. The use of a colonoscopy allows for an even distribution of beneficial bacteria in the stool throughout the colon. However, neither intubation nor mechanical physical invasive methods are considered to be convenient. When administering NG tubes, the risks of vomiting and aspiration should be paid attention to; during colonoscopy, in addition to the increased risk of perforation,^120^ attention should also be paid to the possibility of intestinal bleeding, cardiovascular and cerebrovascular accidents, fever or infection. Colonic TET appears to be a safer and practical method of administration that avoids endoscopic‐related perforations by draining the air and fluid in the colon.^124^ Due to the small size and formula, capsules are more tolerated and convenient than intranasal administration and are more acceptable to patients. When freeze‐dried fecal material is used, capsules can be stored for a long time to maintain a stable supply, making the application more flexible. However, they are also relatively costly, and production of the capsule formulation may be difficult to sustain because of the relatively small number of cases in the current.^125^ Figure 1 provides the general procedures for FMT.

Although FMT has been extensively tested with promising results as described above (Tables 1 and 2), challenges remain in its incorporation into clinical practice. There is a lack of uniform optimal dosing standard for FMT, and its optimal implementation is controversial as mentioned earlier, with some reports suggesting that patients may benefit from multiple series of FMT procedures rather than a single one.^126^ In addition, the consensus clearly states that: “The decision on bowel lavage before microbiota delivery should be considered based on patients' conditions”.^127^ Depending on the recipient's age, immune function, primary diseases and other factors, the efficacy of FMT appears to be complex and uncertain, and different adverse effects may occur. The most commonly reported side effects of FMT are mild reactions, such as abdominal distension, increased defecation, and transient geothermal, and so forth, most of which could be gradually eliminated during follow‐up.^128^, ^129^ Serious adverse events such as death were rare but have been reported.^130^

**TABLE 1:** Changes of gut microbiota before and after FMT treatment.

Disease	Before FMT	After FMT	References
UC	*Clostridium* group XI and Va ↓	Bacteroidota, Bacillota, butyrate‐producing *Faecalibacterium prausnitzii* ↑	20, 28
Colitis	*Clostridium*, gamma *Proteobacteria* ↑	Akkermansia, *Escherichia*, *Blautia*, *Bifidobacterium*, *Collinsella*, Alpha diversity↑ *Bacteroidia*, *Verrucomicrobiae* ↓	33, 34
Constipation	*Enterobacteriaceae*, *Staphylococcus aureus*, fungi ↑ *Bifidobacterium*, *Clostridium*, Bacteroidota (specifically *Prevotella*) ↓		35, 36
MS		*Coprococcus*, *Ruminococcus* ↓	43
Diabetes	Pathogenic *Enterobacteriaceae* ↑ *Butyricogenic* bacteria ↓	Butyrate‐producing bacteria, *Bifidobacterium*, *Lactobacillus* ↑ Anaerobes ↓	49, 52, 53, 58
HE	*Enterococcaceae*, S*taphylococcaceae*, *Enterobacteriaceae*, Streptococcaceae, Ammonia‐producing bacterial ↑ *Clostridium XIV*, Ruminococcaceae, *Lachrillaceae*, *Trichomoniaceae* ↓	*Ruminococcaceae*, *Bifidobacteriaceae* ↑ *Streptococcaceae*, *Veillonellaceae* ↓	65, 67, 68, 72
Hypertension	Bacillota/Bacteroidota ratio ↑ Acetic, butyric and lactic acid‐producing bacteria ↓		75
Cancer	*Eggerthella lenta* ↑ *Parabacteroides distasonis*, *Lactobacillus* ↓	*Veillonellaceae*, *Trichosporonaceae* ↑ *Bifidobacterium* ↓	88, 95
Neurological disorders	Desulfobacterota (proteobacteria), *Uricales*, *Enterobacterales* ↑ Bacillota, *Clostridia* ↓	*Bacteroides*, *Faecalibacterium prausnitzii*, ↑ *Eubacterium coprostanoligenes* ↓	102, 107, 109, 112
Arthritis	*Verrucomicrobiae*, *Akkermansia* ↑		117

**TABLE 2:** The impact of fecal microbiota transplantation on diseases.

Disease	Species	Study design	Results	References
Ulcerative colitis	Human	Randomized clinical trial	Improvement of the gut microbiota.	28
	Human	Prospective cohort study	At week 12, the rate of the primary outcome of clinical and endoscopic remission was higher in the FMT group (54.8%) than in the control group (48.3%)	29
	Human	Randomized controlled trial	Multi‐donor FMT‐AID was valid in leading to deep relief of mild and moderate UC, which was sustained for over 1 year under the anti‐inflammatory diet.	30
ICI‐associated colitis	Human	Observational study	After 3 consecutive FMT treatments, the patient was rapidly relieved, and the symptoms such as diarrhea and gastrointestinal bleeding disappeared completely.	32
	Human	Observational study	FMT improved refractory ICI‐associated colitis by modifying the gut microbiome.	33
	Human	Observational study	FMT effectively treat patients with immune‐mediated colitis with a significant increase in alpha diversity and increases in the abundances of *Collinsella* and *Bifidobacterium*.	34
Constipation	Human	Observational study	Improved efficacy of FMT in slow transit constipation.	38
	Human	Randomized, double‐blind controlled trial	FMT could enhance the efficacy of retrograde colonic enema.	39
Metabolic syndrome	Rat	Animal experiment	FMT reduced markers of oxidative stress and inflammation.	43
	Mice	Animal experiment	FMT counteracted high fat diet‐induced weight gain and epididymal visceral adipose tissue mass, inflammation‐related gene expression, insulin resistance, and glucose tolerance.	44
	Human	Randomized, double‐blind, placebo‐controlled phase 2 trial	Single‐dose oral FMT transplantation to patients with metabolic syndrome was safe and well tolerated and also improved insulin sensitivity.	5
	Human	Randomized, double‐mashed, placebo‐controlled trial	FMT improved insulin sensitivity and glucose metabolism.	45
	Human	Retrospective study	WMT improved the metabolic characteristics of MS patients, downgraded ASCVD risk in the high‐risk group of patients with MS and restored gut microbiota homeostasis in MS patient.	46
Diabetes	Mice	Animal experiment	FMT improved insulin resistance, repaired damaged islets, inhibited apoptosis of islet β cells and restored gut microbiota.	51
	Human	24‐week, double‐blind, randomized controlled trial	Repeated FMT enhanced the efficacy and duration of efficacy in T2DM patients.	52
	Human	Prospective cohort study	FMT improved the symptoms of T2DM.	53
	Human	Observational study	FMT stabilized residual β‐cell function in subjects with new‐onset T1D and prevented the decline in endogenous insulin production.	56
	Human	Observational study	The patient had a good clinical response after FMT.	57
	Mice	Animal experiment	FMT markedly reduced blood glucose and augmented *Lactobacillus* species in the small intestine.	58
	Human	Randomized, double‐blind, and placebo‐controlled trial	FMT significantly ameliorated distal symmetric polyneuropathy.	59
	Human	Single‐arm, self‐controlled clinical trial	FMT significantly ameliorated glycemic variability.	60
Hepatic encephalopathy	human	Observational study	Persistent FMT resulted in cognitive improvement in mild HE.	70
	Human	Phase 1, Randomized, Placebo‐Controlled Trial	Oral FMT capsules were safe and tolerated in cirrhosis and recurrent HE patients.	72
Hypertension	Rat	Animal experiment	FMT reduced BP.	79
	Human	Retrospective study	WMT showed a short‐term (3–7 days) but not medium‐term (1 month) hypotensive function in hypertensive subjects, but its duration of action was longer than that of conventional antihypertensive drugs.	84
Cancer	Human	Observational study	FMT treatment altered immune cell infiltration and gene expression profiles in the gut lamina propria and tumor microenvironment.	95
	Human	Observational study	FMT and anti‐PD‐1 together successfully colonized the gut of responders and altered the gut microbiota composition, reprogramming the tumor microenvironment.	96
	Human	Open‐label, single‐arm, phase II trial	FMT showed good anti‐tumor efficacy and acceptable safety for the treatment	97
Neurological disorders	Mice	Animal experiment	FMT alleviated “plaque disruption” and behavioral changes.	100
	mice	Animal experiment	FMT treatment ameliorated cognitive deficits and reduced brain Aβ deposition.	101
	Mice	Animal experiment	FMT inhibited intestinal and neural inflammation and suppressed TLR‐4/TNF‐α signaling.	102
	Human	Randomized repeat‐dose, placebo‐controlled clinical pilot study	FMT reduced constipation, improved bowel transit, bowel motility, subjective motor and non‐motor symptoms.	103
	Human	Nonrandomized, open‐label study	FMT improved GI symptoms and ASD‐related behaviors.	106
	Human	Open‐label study	FMT altered gut microbiota.	107
	human	Observational study	FMT improved depressive symptoms and gastrointestinal symptoms.	109
	Human	Observational study	WMT prevented amyotrophic lateral sclerosis from worsening.	111
	Human	Observational study	FMT ameliorated respiratory failure in amyotrophic lateral sclerosis.	112
	Human	Phase 2, single center, randomized clinical trial	FMT improved motor and non‐motor symptoms.	113
Arthritis	Human	Observational study	FMT improved AS symptoms.	119
	Human	Observational study	FMT reduced Health assessment questionnaire disability index, disease activity score, rheumatoid factor titer, and medication dose.	118
	Human	Randomized controlled trial	FMT may induce a systemic immune response to PsA.	16

To standardize the development of FMT, the laboratory preparation of fecal microbiota has been modified from the traditional FMT method to WMT, designed to deliver precise doses of microbiota.^131^ In addition, a 1‐h FMT protocol (reduced to 1 h from defecation to infusion or defecation to freezing) has also been implemented to improve the content of live stool microorganisms and the applicability of clinical stool materials.^132^ A stepwise strategy for FMT has been further proposed: Step 1 refers to a single FMT, Step 2 refers to multiple FMT (≥2 times) and Step 3 refers to the combination of FMT with conventional medical therapy after the failure of Step 1 or Step 2, with the aim of advancing the success rate of FMT and reducing the incidence of adverse events after FMT.^133^ In response to the scrutiny of FMT safety and efficacy, the American Gastroenterological Association has set up a national FMT registry designed to assess the short‐ and long‐term safety of FMT and other gut‐associated microbiota products by collecting clinical data up to 10 years after FMT.^134^ Likewise, China, Australia, the United Kingdom, and other countries have also issued relevant monitoring documents.^135^, ^136^, ^137^, ^138^

## 5 CONCLUSION

Gut microbiota is well known to be closely related to human health. Identifying its components is the first step to a personalized diagnostic approach, and understanding the individual gut microbial community alterations during the course of the disease lays the foundation for personalized treatment. FMT holds great potential for curing disease, and in recent years it has evolved into an increasingly standardized approach, attracting a great many research teams to conduct clinical trials. These theoretical research results and clinical application practice provide reference for the establishment of a standard FMT framework. However, the specificity of the disease response to FMT, the optimal dose and frequency of FMT, and whether the response is repeatable and long‐lasting remains to be answered. Most damningly, most of the current evidence is based on small samples. Therefore, high‐quality, large‐scale controlled trials are still needed to thoroughly investigate the mode of action, effective therapy concentrations, potential adverse events, reasonable cost control and long‐term consequences of FMT before it can be widely used in clinical practice.

## AUTHOR CONTRIBUTIONS

**Dongxin Yi:** Methodology; validation; writing – review and editing; writing – original draft; investigation. **Tao Li:** Investigation; validation; conceptualization. **Yuji Xiao:** Conceptualization; investigation; validation. **Xue Zhang:** Conceptualization; investigation; validation. **Qiangqiang Hao:** Conceptualization; investigation; validation. **Feng Zhang:** Conceptualization; investigation; validation. **Tianming Qiu:** Conceptualization; investigation; validation. **Guang Yang:** Conceptualization; investigation; validation. **Xiance Sun:** Conceptualization; investigation; validation. **Ying Dong:** Resources; funding acquisition; supervision. **Ningning Wang:** Supervision; funding acquisition; resources.

## FUNDING INFORMATION

This work is supported by the National Natural Science Foundation of China (Grant No. 82003476), and the Dalian Medical University Interdisciplinary Research Cooperation Project Team Funding (Grant No. JCHZ2023012).

## CONFLICT OF INTEREST STATEMENT

The authors declare no conflicts of interest.

### PEER REVIEW

The peer review history for this article is available at https://www.webofscience.com/api/gateway/wos/peer-review/10.1002/btm2.10728.

## Data Availability

Data sharing is not applicable to this article as no new data were created or analyzed in this study.
